# A Marked Effect of Electroconvulsive Stimulation on Behavioral Aberration of Mice with Neuron-Specific Mitochondrial DNA Defects

**DOI:** 10.1371/journal.pone.0001877

**Published:** 2008-03-26

**Authors:** Takaoki Kasahara, Mie Kubota, Taeko Miyauchi, Mizuho Ishiwata, Tadafumi Kato

**Affiliations:** Laboratory for Molecular Dynamics of Mental Disorders, Brain Science Institute, RIKEN, Wako-shi, Saitama, Japan; University of Florida, United States of America

## Abstract

We developed transgenic (Tg) mice modeling an autosomally inherited mitochondrial disease, chronic progressive external ophthalmoplegia, patients with which sometimes have comorbid mood disorders. The mutant animals exhibited bipolar disorder-like phenotypes, such as a distorted day–night rhythm and a robust activity change with a period of 4–5 days, and the behavioral abnormalities were improved by lithium. In this study, we tested the effect of electroconvulsive stimulation (ECS) on the behavioral abnormalities of the model. Electroconvulsive therapy, which has long been used in clinical practice, provides fast-acting relief to depressive patients and drug-resistant patients. We performed long-term recordings of wheel-running activity of Tg and non-Tg mice. While recording, we administrated a train of ECS to mice, six times over two weeks or three times over a week. The treatment ameliorated the distorted day–night rhythm within three times of ECS, but it had no effect on the activity change with a period of 4–5 days in the female mice. To study the mechanism of the action, we investigated whether ECS could alter the circadian phase but found no influence on the circadian clock system. The potent and fast-acting efficacy of ECS in the mutant mice supports the predictive validity of the mice as a model of bipolar disorder. This model will be useful in developing a safe and effective alternative to lithium or electroconvulsive therapy.

## Introduction

Bipolar disorder is a major mental disorder characterized by recurrent manic and depressive episodes. The involvement of genetic factors in bipolar disorder has been recognized from twin, adoption, and family studies [1]. Although linkage analyses and case-control association studies have been conducted, robustly replicated loci susceptible to bipolar disorder have not been identified [2], [3]. In this context, several symptom-based animal models have been reported [4]–[6]. But such models hardly satisfy the construct validity; that is, these animals do not mimic aspects of the etiology.

Our group has focused on a single major gene defect with pleiotropic effects, one of which manifests bipolar disorder. Patients with chronic progressive external ophthalmoplegia (CPEO) are sometimes affected with comorbid mood disorders [7]. CPEO is a rare disease (MIM 157640) inherited in a Mendelian fashion. It is characterized by slowly but progressive ptosis and ophthalmoparesis and is associated with mitochondrial DNA (mtDNA) aberrations such as deletions, duplications, and point mutations. Mitochondrial dysfunction in bipolar disorder has been suggested by magnetic resonance spectroscopy, association studies of polymorphisms of mtDNA and nuclear genes encoding mitochondrial protein, and genome-wide gene expression analyses [8]. Recently, we generated transgenic mice as a model of bipolar disorder, in which mutant POLG (mtDNA polymerase) was expressed in a neuron-specific manner [7]. The *POLG* gene is reported to be one of the causative genes for CPEO comorbid with mood disorders [9].

The transgenic mice (mutPOLG Tg mice) harbor mtDNA defects in the brains, which show altered activities of serotonin and noradrenalin. When the mutant animals are subjected to voluntary wheel running, they show distorted day–night rhythm; they exhibit continued activity after the light is turned on (delayed activity) and untimely activity before the light is turned off (anticipatory activity). In addition, female mutPOLG Tg mice show a robust periodic activity pattern associated with the estrous cycle. These behavioral abnormalities are improved by lithium. Therefore, we propose that mutPOLG Tg mice satisfy the construct, face, and predictive validities of a model for bipolar disorder [7].

Our group has begun to pursue the cellular pathomechanism of mutPOLG Tg mice [10], as the model would accelerate medication development as well as basic research. Prior to medication development using this model, we focused on electroconvulsive therapy (ECT) in this study. ECT has long been used since before the discovery of lithium's efficacy as a mood-stabilizing agent, and ECT provides fast-acting relief to depressive patients and drug-resistant patients [11]. Here we report that electroconvulsive stimulation (ECS) markedly and immediately ameliorated the behavioral aberration of mutPOLG Tg mice.

## Results

### The effect of ECS on the behavioral phenotypes of mutPOLG mice

**Figure 1:**
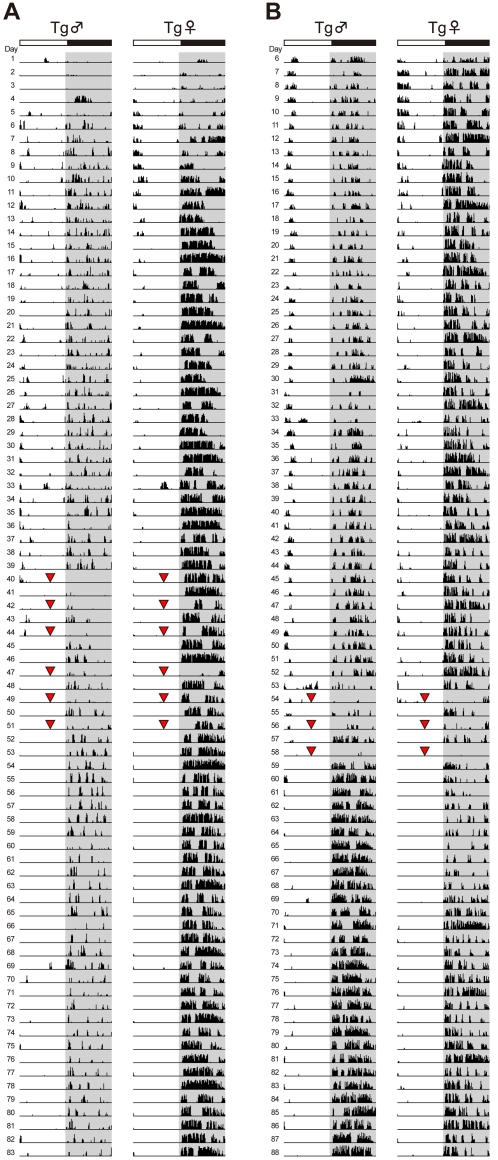
Wheel-running activity records of representative mice receiving ECS. Mice were delivered ECS (red triangles) six times (A) or three times (B). The light and dark periods (12:12 h) are indicated by white and gray backgrounds, respectively.

We delivered ECS six times over 2 weeks in the first experiment (Fig. 1A); this treatment schedule conformed to the standard ECT regime for patients with mood disorder. ECS seemed to improve the distorted day–night rhythm of the mutPOLG Tg mice; however, more than half of the treated mice died after ECS probably due to respiratory failure, and it became impossible to statistically analyze the effect of ECS. Therefore, in the second experiment, mice breathed in oxygen gas for about 1 min before receiving the electroshock. Oxygenation was highly effective, and mice rarely died after ECS.

**Figure 2:**
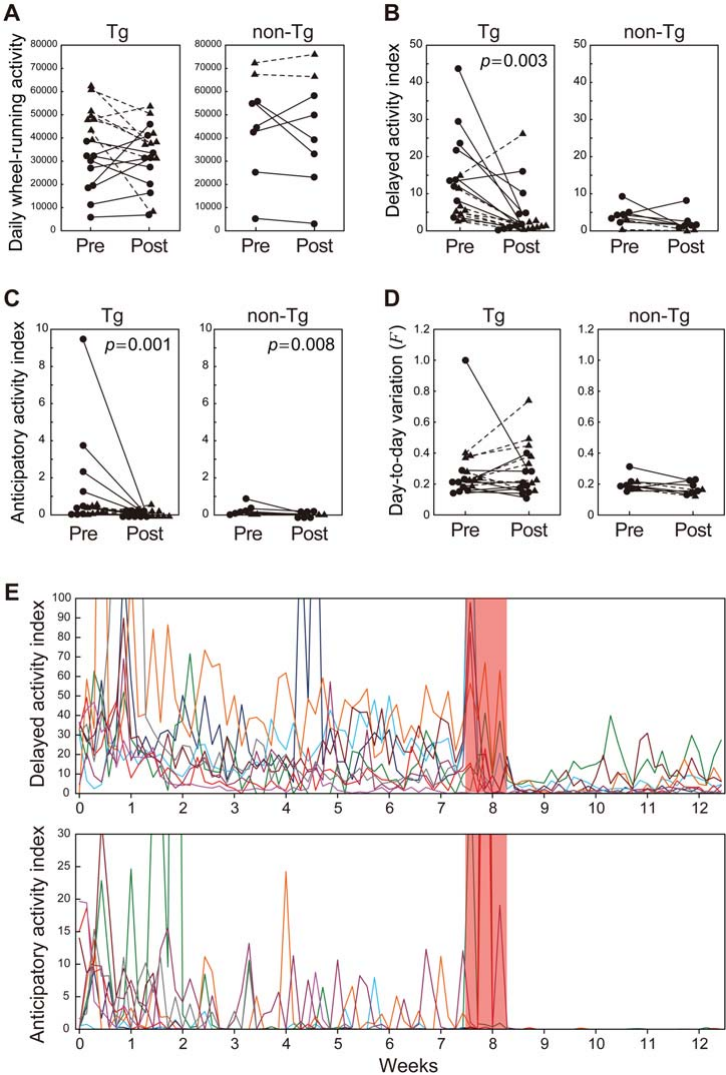
Effect of ECS. Mice were oxygenized and delivered ECS three times within a week (days 54, 56, and 58; shown in Fig. 1B). Wheel-running activity (A), delayed (B) and anticipatory (C) activity indices, and day-to-day variation in activity (D) were estimated on the basis of the activity levels before (Pre) and after (Post) the treatment (days 11–53 and 66–89, respectively). Values from individual male mice are indicated by closed circles connected with solid lines, and values from individual female mice are indicated by closed triangles connected with dashed lines. Delayed activity index of Tg mice and anticipatory activity index of both Tg and non-Tg mice were significantly lowered by ECS. (E) Time courses of delayed and anticipatory activity indices of male Tg mice receiving ECS. Each color of line represents different individuals (*n* = 9). The week when three ECS treatments were delivered is indicated by a red background.

Because effects of ECS were observed within the first week (after three treatments) in the first experiment, we delivered ECS three times over 1 week in the second experiment (Fig. 1B). ECS caused a reduction in wheel-running activity in the night following ECS, but this returned to the baseline levels after 1 week (Figs. 1B and 2A). Delayed and anticipatory activities, which are indicators of distorted day–night rhythm, were markedly improved (Fig. 2B and C). However, the robust periodic activity change observed in female Tg mice and day-to-day variation in wheel-running activity, one indicator of the periodic activity change, were not alleviated by ECS (Figs. 1 and 2D). Both delayed and anticipatory activities were suppressed for about 1 week after ECS (Fig. 2E), and some mice subsequently exhibited relapse of the delayed activity. In contrast, the anticipatory activity was strictly suppressed until the end of the experiment. ECS did not improve the robust periodic activity change of the female Tg mice (Fig. 1).

### No influence of ECS on the phase of circadian clock

**Figure 3:**
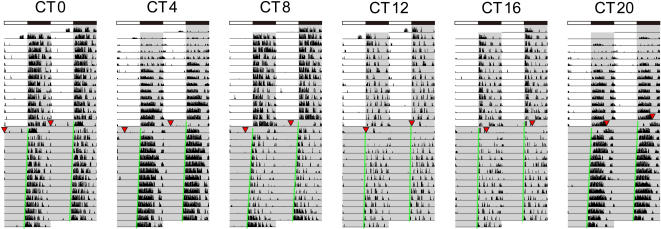
No influence of ECS on the circadian clock. Double-plotted activity records of representative mice receiving one ECS (red triangles) at different times of the day: circadian time (CT) 0, 4, 8, 12, 16, and 20. CT0–12 is the subjective light phase, and CT12–24 is the subjective dark phase. The light and dark periods are indicated by white and gray backgrounds, respectively. Onset of activity shown by green lines was used as the reference phase for the determination of phase shift.

In some cases, ECS-treated mice showed regular day–night rhythm soon after the first ESC. The immediate effect of ECS allowed us to assume that ECS induced phase shifts of the circadian clock and adjusted the day–night rhythm. ECS as well as transcranial magnetic stimulation induced *c-fos* mRNA expression in the suprachiasmatic nucleus (SCN) [12], where the master circadian clock located. It is also known that *c-fos* expression is coincident with light-induced phase-shift of the circadian clock [13], [14]. Thus, it might be possible that ECS improved the phenotype of the mice by directly affecting the SCN. To test this hypothesis, we examined whether ECS could alter the phase of circadian clock system. Animals were transferred and maintained in constant darkness, resulting in the free-running of circadian clock. Then we delivered a single ECS under dim red light at various times of day. We observed no phase shift caused by the ECS at any time of the day (Fig. 3).

## Discussion

ECS had a dramatic effect on mutPOLG Tg mice. Delayed and anticipatory activities, indices of distorted day–night rhythm, were markedly improved by ECS (Fig. 2B, C and E). This rapid and potent effect of ECS is quite similar to that of ECT for patients with mood disorder. ECT is regarded as the most effective treatment for depression and mania [11]. According to several reports, patients with bipolar disorder respond more rapidly to ECT and require fewer treatments than unipolar patients [15], [16]. In mutPOLG Tg mice, three treatments of ECS sufficiently improved both delayed and anticipatory activities. We observed the wheel-running activity of ECS-treated Tg mice for one month and found two significant outcomes. First, delayed activity suppressed by ECS gradually relapsed about 2 weeks afterward (Fig. 2E, upper), whereas anticipatory activity was completely suppressed over the 1-month period (Fig. 2E, lower). These findings suggest that there might be different mechanisms underlying delayed and anticipatory activities. Second, a robust activity change associated with the estrous cycle was not improved by ECS (Figs. 1 and 2D). Since lithium improved the periodic activity change as well as the distorted day–night rhythm [7], we had hypothesized that the two phenotypes of the mutPOLG mice were closely related. The present result, however, indicate that these two phenotypes have different treatment response.

The potent and fast-acting efficacy of ECS in the mutant mice supports the predictive validity of the mice as a model of bipolar disorder. Although the molecular mechanism underlying the actions of lithium and ECT/ECS remains unclear, neurotrophic growth factors are suggested to be upregulated by lithium [17], [18] and ECS [19]–[21]. This model will be useful in elucidating the action mechanism of these medications and also in developing a safe and effective alternative to lithium or ECT.

## Materials and Methods

### Animals

All the mutant POLG Tg mice used were heterozygotes. Male mutant mice were used for mating to avoid possible transmission of mtDNA mutations from the maternal side, although expression of the transgene is restricted in neuronal tissues. Genomic DNA was isolated from tail biopsies, and the genotyping was performed by multiplex PCR using the two sets of primers: Fw, 5′-TGG TGA AAC AGT TGA ATC TTC C-3′; Rv, 5′-GTC AGG AGA TTG GTG ATC TGC-3′; and Fw, 5′-AGT GAG TTG AAA GCC ATG GTG-3′; Rv, 5′-GTG GTT GAA CTG CAT CAG TAG G-3′. Controls were non-Tg littermates whenever possible. Mice of older than 26 weeks of age were used to measure wheel-running activity, because mtDNA defects were not accumulated in the young mutPOLG Tg mice [7]. The Wako Animal Experiment Committee, RIKEN, approved all animal experiment protocols.

### Long-term recording of wheel-running activity and statistic analyses

Recoding and analyses of wheel-running activity were performed as described previously [7]. Wheel-running activity (three counts per rotation) was recorded every 10 min by an online PC computer system (O'Hara & Co., Tokyo, Japan).

The delayed activity index is defined as a percent of the activity during the first 3 h of the light period divided by the total activity during the previous dark period (12 h). Anticipatory activity index is defined as a percent of the activity during the last 3 h of the light period divided by the activity during the following dark period (12 h). Day-to-day variation in wheel-running activity was estimated by the unevenness in daily wheel-running activity, which is the total number of counts of wheel running per day. The equation used to calculate day-to-day variation was described in our previous report [7]. Wilcoxon signed-rank tests were used to test for any difference in these indices before versus after the treatment. Values of *p* < 0.05 were considered statistically significant.

### Electroconvulsive stimulation

Animals were handled on the day before the first ECS to become adjusted to immobilization in a small cylinder and the application of ear-clip electrodes. Ear-clip electrodes were dampened with saline and attached to the pinnae, and electroshock (12–50 mA, 60 pulses/sec, 0.5-msec pulse width, 1-sec duration) was delivered using an electroconvulsive device (Ugo Basile, Comerio, Italy) and a bipolar square-wave pulse generator (Ugo Basile). A tonic seizure was characterized by extension of all limbs and forward head extension that lasted for about 10 sec. We titrated the dose (current amplitude) upward in 2-mA steps beginning at 10–15 mA, which depended on body weight. Because repeated ECS increased the seizure threshold, we administered ECS of the last threshold intensity, and we titrated the intensity upward if the animal did not exhibit a tonic seizure. Mice were treated 6 times in 2 weeks in the first experiment (male Tg, *n* = 8; male non-Tg, *n* = 7, 37–48 (44.6±3.8) weeks old at the start of recording; female Tg, *n* = 8; and female non-Tg, *n* = 7, 37–53 (44.3±5.6) weeks old) and 3 times in 1 week in the second experiment (male Tg, *n* = 9; male non-Tg, *n* = 6, 32–50 (42.8±7.1) weeks old; female Tg, *n* = 8; and female non-Tg, *n* = 2, 26–48 (40.0±6.8) weeks old). In the second experiment, to prevent death due to respiratory failure, we placed mice in a plastic bag filled with oxygen gas for about 1 min before delivering the electroshock. The oxygen gas was supplied to mice after ECS until they began breathing regularly. To investigate the influence of ECS on the circadian clock, in a third experiment we delivered a single ECS at various times of day under dim red light. The mice were placed in constant darkness before delivering ECS. The light conditions were shown in Fig. 3.
